# One-step large-scale synthesis of micrometer-sized silver nanosheets by a template-free electrochemical method

**DOI:** 10.1186/1556-276X-8-248

**Published:** 2013-05-22

**Authors:** Sun Hwa Park, Jin Gyeong Son, Tae Geol Lee, Hyun Min Park, Jae Yong Song

**Affiliations:** 1 University of Science and Technology, Daejeon 305-350, Republic of Korea; 2 Korea Advanced Institute of Science and Technology, Daejeon 305-701, Republic of Korea; 3 Korea Research Institute of Standard and Science, Daejeon 305-340, Republic of Korea

**Keywords:** Silver, Nanosheets, Template free, Electrochemical deposition

## Abstract

We have synthesized micrometer-sized Ag nanosheets via a facile, one-step, template-free electrochemical deposition in an ultra-dilute silver nitrate aqueous electrolyte. The nanosheet growth was revealed to occur in three stages: (1) formation of polygonal Ag nuclei on a substrate, (2) growth of {112}-faceted nanowire from the nuclei, and (3) anisotropic growth of (111)-planar nanosheets, approximately 20 to 50 nm in thickness and 10 μm in width, in the <112>−direction. The vertical growth of the facet nanowire was induced by the strong interface anisotropy between the deposit and electrolyte due to the ultra-dilute concentration of electrolyte and high reduction potential. The thickness of Ag nanosheets was controllable by the adjustment of the reduction/oxidation potential and frequency of the reverse-pulse potentiodynamic mode.

## Background

Silver nanostructures have attracted much attention due to unique electrical, optical, and biocompatible properties that are applicable to chemical sensors, catalysts, interconnects in micro or nano devices, plasmonics, and photonics [1-5]. The chemical properties of Ag nanostructures are determined by their morphology, size, crystallographic plane, and alloying composition [6-8]. Among various silver nanostructures, nanoplates or nanosheets, particularly, have been intensively investigated because they have the size- and shape-sensitive surface plasmon resonance bands [1,8-12].

Until now, two-dimensional silver nanostructures have been fabricated using surfactants (capping agent) [6,13], sacrificial materials [14], and hard templates (porous alumina) [15]. Although these methods have the merits of controlling the morphology and size of Ag nanostructures, they are complicated and costly. A chemical route without any surfactants led to the large-scale synthesis of micrometer-sized Ag nanosheets (approximately 15 μm in size and 28 nm in thickness) after the addition of a small quantity of H_2_PdCl_4_ as seeds for the growth of Ag nanosheets [16]. With such solution-based methods, colloidal nanosheets were randomly dispersed in a liquid before being used for their purposes. Recently, a simple galvanic reaction was used to synthesize silver nanoplates with thicknesses of 50 to 70 nm and a size of approximately 1 μm on GaAs substrates [9,17]. In an alternative approach, current density of a potentiostatic electrochemical method using poly(vinyl pyrrolidone) was kinetically controlled to synthesize vertically cross-linking Ag nanosheets of several micrometers in width [8,18]. However, there are very limited studies on the facile and large-scale synthesis of Ag nanosheets by an electrochemical deposition without any templates and surfactants.

In this study, we report a facile, large-scale, one-step process of synthesizing Ag nanosheets (tens of micrometers in size and several tens of nanometers in thickness). Our process uses a template- and surfactant-free electrochemical deposition in an ultra-dilute electrolyte of low electrical conductivity (less than 50 μS∙cm^−1^). The growth mechanism was revealed by time-dependent growth analyses. The present method is environment friendly and low cost because the precursor concentration of Ag ions is very low (several tens of μM) compared with that (above several mM) used in conventional electrochemical methods.

## Methods

### Preparation of Ag nanosheets

Ag nanosheets were deposited on a substrate by a reverse-pulse potentiodynamic electrochemical deposition. The aqueous electrolyte was composed of 0.02 mM AgNO_3_ (#209139, reagent A.C.S., Sigma-Aldrich, St. Louis, MO, USA) and 1.32 mM NH_4_OH (#13370-0380, Guaranteed Reagent, Junsei Chemical Co., Ltd., Chuo-ku, Tokyo, Japan). The AgNO_3_ concentration was varied as 0.2 and 2 mM, respectively, to observe the effects of concentration on the morphologies of Ag deposits. A two-electrode system that comprised a Ag plate (1 mm in thickness and 5 cm in length, 99.9%, Alfa Aesar, Wardhill, MA, USA) as a counter electrode and a Au film-coated Si substrate as a working electrode was used. The exposed area of Au film (90-nm thick) was 0.5 cm × 0.5 cm. The electrolyte was supplied into the rectangular Teflon bath at the constant flow rate of 200 ml/min using a peristaltic pump (# S 600, dslab 24, Gyeonggi-do, Korea). The interdistance between the working and counter electrodes was set at 1 cm. For the reverse-pulse potentiodynamic mode, the reduction potentials (*V*_R_) were set to be 10, 15, and 20 V, and oxidation potentials (*V*_O_) were set to be 0.05, 0.2, and 0.4 V. The deposition time was varied as 20, 40, 70, and 120 min, respectively. The frequency was controlled as 1, 10, 100, and 1,000 Hz, respectively. The reduction period of the reverse-pulse was set at 3%.

### Instruments and characterization

The homemade two-electrode system was composed of a dual DC power supply (Agilent E3620A, Agilent Technologies, Santa Clara, CA, USA) and a function generator (Agilent 33220A). The detailed description can be found in previous work [19]. The microstructures of Ag nanosheets were observed using a field-emission scanning electron microscope (SEM; Hitachi S-4800, Hitachi Ltd., Chiyoda-ku, Japan). The crystal structures were analyzed using a high-resolution transmission electron microscope (HRTEM; FEI Tecnai G2 F30, 300 kV, FEI, Hillsboro, OR, USA).

## Results and discussion

**Figure 1:**
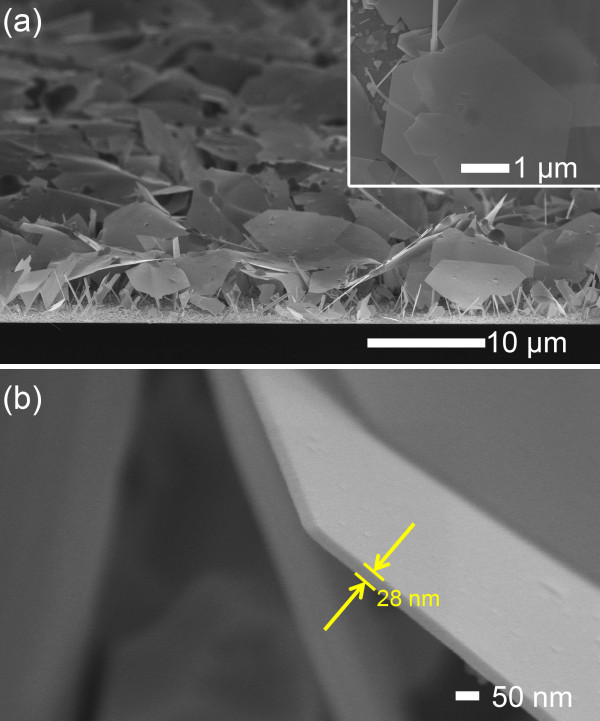
**Typical SEM images of Ag nanosheets.** (**a**) Typical 13°-tilted SEM images of Ag nanosheets grown on a substrate and (**b**) a higher magnified SEM image of a Ag nanosheet. (The inset indicates a higher magnified top-view SEM image.).

**Figure 2:**
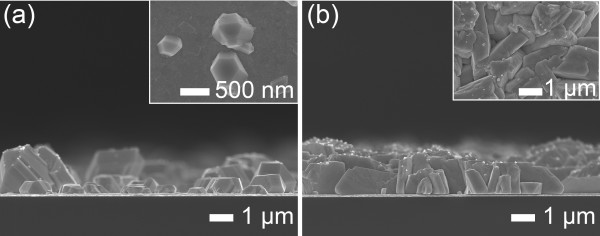
**Typical SEM images of Ag deposits with AgNO**_**3 **_**concentration.** Cross-sectional SEM images of Ag deposits deposited in the electrolytes of (**a**) 0.2 and (**b**) 2 mM AgNO_3_ for 120 min (*V*_R_ = 15 V, *V*_O_ = 0.2 V, 100 Hz, and 3%). (The insets denote the top-view SEM images.).

Figure 1 shows the typical SEM images of Ag nanosheets that were electrodeposited in an ultra-dilute electrolyte in the potentiodynamic mode (*V*_R_ = 15 V, *V*_O_ = 0.2 V, 100 Hz, and 3%) for 120 min. Ag nanosheets had a width up to approximately 10 μm and a thickness of approximately 30 nm and were grown on the facetted Ag nanowires. In comparison, when the AgNO_3_ concentration was 0.2 mM, the facetted granular Ag islands grew with the size of 0.2 to 2 μm, as shown in Figure 2a. With the further increase of AgNO_3_ concentration up to 2 mM, the granular islands were densely generated and formed a granular (columnar) layer, as shown in Figure 2b. This indicates that the growth of facetted nanowires and nanosheets shown in Figure 1 was closely related to the dilute concentration.

**Figure 3:**
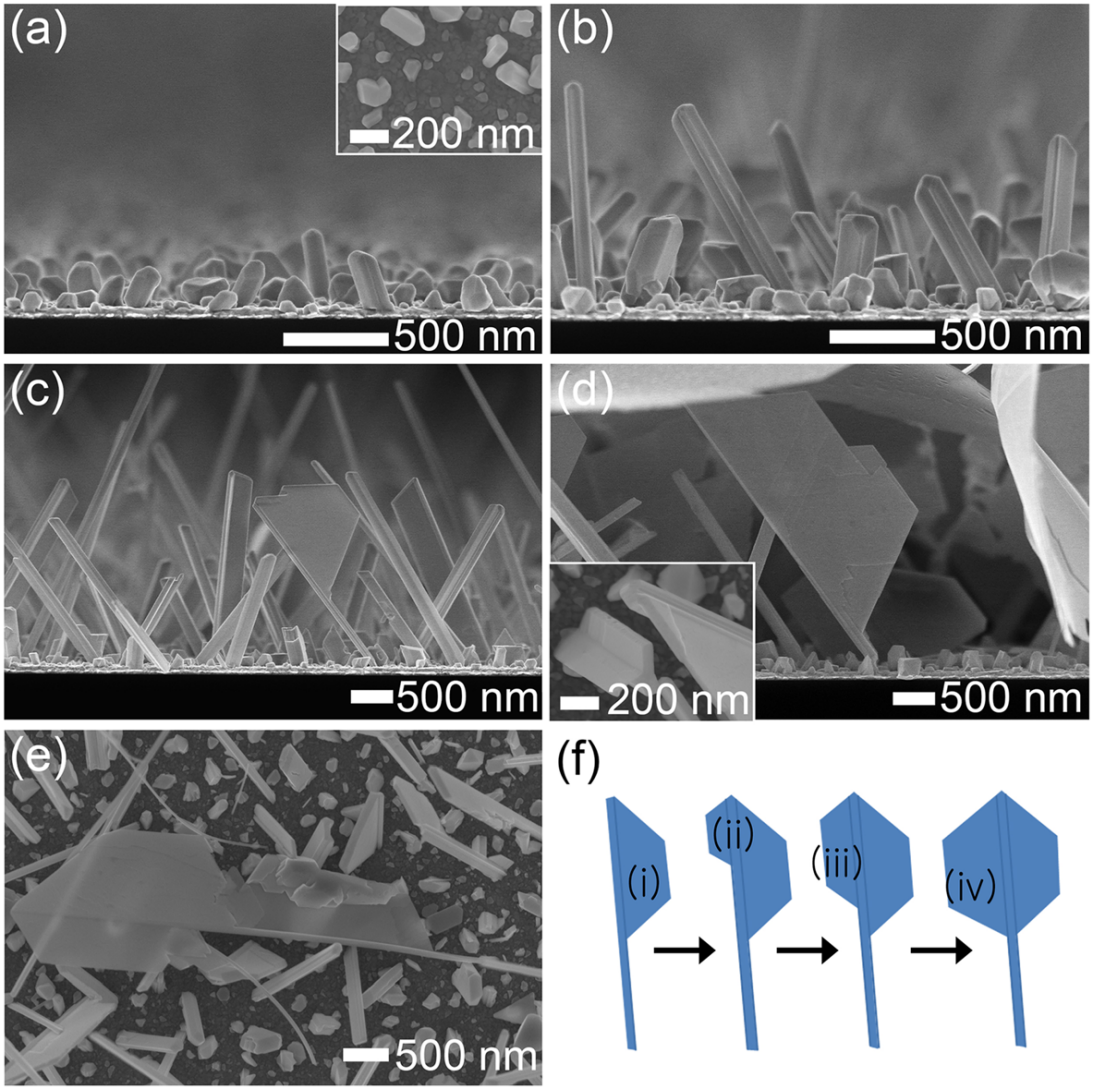
**Time-dependent growth morphology of Ag nanosheets.** Cross-sectional SEM images of Ag nanosheets with deposition times of (**a**) 20, (**b**) 40, (**c**) 70, and (**d**) 120 min. (**e**) Enlarged top-view SEM image of the specimen shown in (**c**). (**f**) Schematic diagram of illustrating the growth of hexagonal nanosheets. (The insets denote the top-view SEM images.).

The time-dependent growth of the Ag nanosheets was examined by varying the deposition time as 20, 40, 70, and 120 min, respectively, as shown in Figure 3a,b,c,d. The growth occurred in three stages. First, the nucleation of polygonal islands on a substrate occurred, as shown in Figure 3a. The polygonal nuclei were randomly generated on the whole surface of substrate. Second, one-dimensional growth was driven in a specific direction by strong interface anisotropy between the polygonal islands and the electrolyte, which resulted in the facetted Ag nanowires shown in Figure 3b. In the previous work, it was shown that the interface anisotropy becomes stronger due to the field enhancement at the top of the hemispherical islands in an ultra-dilute electrolyte of low electrical conductivity [20]. Third, planar growth on one of the facet planes was initiated and planar nanostructure grew further, forming a facetted nanosheet (Figure 3c). The nanosheets, which were attached to the facetted nanowires, grew wider (up to approximately 10 μm) with increasing deposition time, as shown in Figure 3d. Figure 3e shows the enlarged top-view SEM image of the nanosheet on the specimen shown in Figure 3c. The growth of hexagonal nanosheet can be described, as shown in Figure 3f. After the planar growth (i) on one facet plane of the facetted nanowire, another planar growth occurs on the other facet plane (ii), as shown in Figure 3e. The nanosheet grows further with deposition time and finally forms a hexagonal nanostructure (iv). The whole surface of the substrate was covered with Ag nanosheets that grew along the facetted nanowires, as shown in Figure 1a. The nanosheets attached to the facetted nanowires could easily be detached from the substrate and dispersed into an aqueous solution via sonication for several seconds, which enabled us to easily prepare TEM samples.

**Figure 4:**
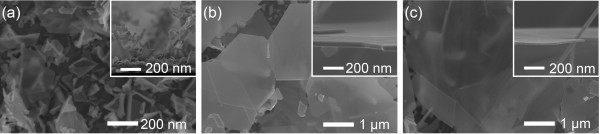
**Controllable thickness of Ag nanosheets.** Top-view SEM images of Ag nanosheets grown at various deposition frequencies of (**a**) 1 Hz, (**b**) 10 Hz, and (**c**) 1 kHz for 120 min. (The insets denote the higher magnified cross-sectional SEM images of Ag nanosheets.).

**Figure 5:**
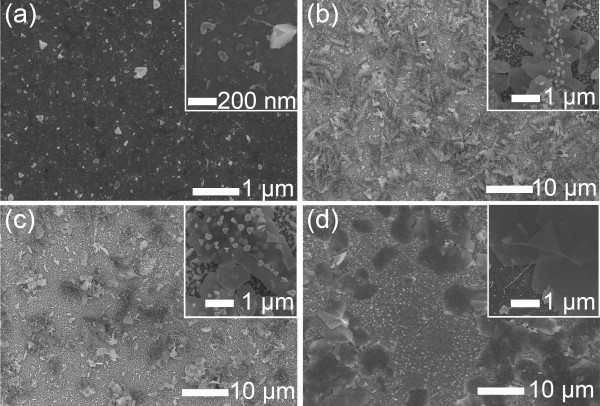
**Morphological variations of Ag nanosheets.** Top-view SEM images of Ag nanosheets grown in the electrolyte composed of 20 μM AgNO_3_ and 1.32 mM NH_4_OH for 120 min. Comparing the deposition condition (*V*_R_ = 15 V, *V*_O_ = 0.2 V, 100 Hz, and 3%) for the sample shown in Figure 1, the reduction potential (*V*_R_) was varied as (**a**) −10 and (**b**) −20 V, and the oxidation potential (*V*_O_) as (**c**) 0.05 and (**d**) 0.4 V, respectively. (The insets are magnified top-view SEM images.).

As shown in Figure 4, the thickness of the nanosheet depended on the thickness of the facetted nanowires that grew over the islands nucleated on the substrate. Therefore, the thickness of Ag nanosheets could be controlled by varying the island size. In the previous work, the island size was controlled by the deposition frequency and reduction/oxidation potentials of the reverse-pulse potentiodynamic mode [20]. When the deposition frequency was varied in the range of 1 to 1,000 Hz under the same deposition parameters (*V*_O_, *V*_R_, and duty) for the sample shown in Figure 1, the thickness and size of Ag nanosheets were controlled in the range of 20 to 50 nm and 3 to 10 μm in size, respectively (Figure 4). At the low frequency of 1 Hz, the deposit was composed of irregular Ag nanosheets shown in Figure 4a. With increase of the frequency from 10 to 1,000 Hz, the planar Ag grew and the thickness decreased from 50 to 20 nm, approximately. Also, the nanosheet size increased with the frequency increasing, as shown in Figure 4. It is noted that the facetted nanowires became thinner with the frequency increasing in the range. It is presumed that the nucleation size became smaller with the shorter period of reduction process. We investigated the effects of the reduction/oxidation potentials on the growth of Ag nanosheets, as shown in Figure 5. At the reduction potential of −10 V (Figure 5a), the deposit grew so slowly comparing to that shown in Figure 1. It seems that the reduction potential should be applied over *V*_R_ = −10 V. At the higher reduction potential of −20 V, a lot of nanosheets were deposited and extra nanoparticles grew on the nanosheet surface, as shown in Figure 5b. This was due to the fact that the higher reduction potential leads to higher nucleation and growth rates in electrochemistry. Also, when the oxidation potential was decreased to 0.05 V comparing with the samples (*V*_O_ = 0.2 V) shown in Figure 1, nanosheets of several micrometers in size grew, and small nanoparticles were deposited on the surface of the nanosheets, as shown in Figure 5c. At the higher V_O_ of 0.4 V, nanosheets grew without nanoparticles on their surface, but the amount of nanosheets decreased much, as shown in Figure 5d. As the oxidation potential played a role of dissolving Ag atoms into the electrolyte, the lower oxidation potential resulted in the higher growth rate, and vice versa [20]. Therefore, the morphology of Ag nanosheets shown in Figure 5c was similar to that of Ag nanosheets which were deposited at the higher reduction potential of −20 V.

**Figure 6:**
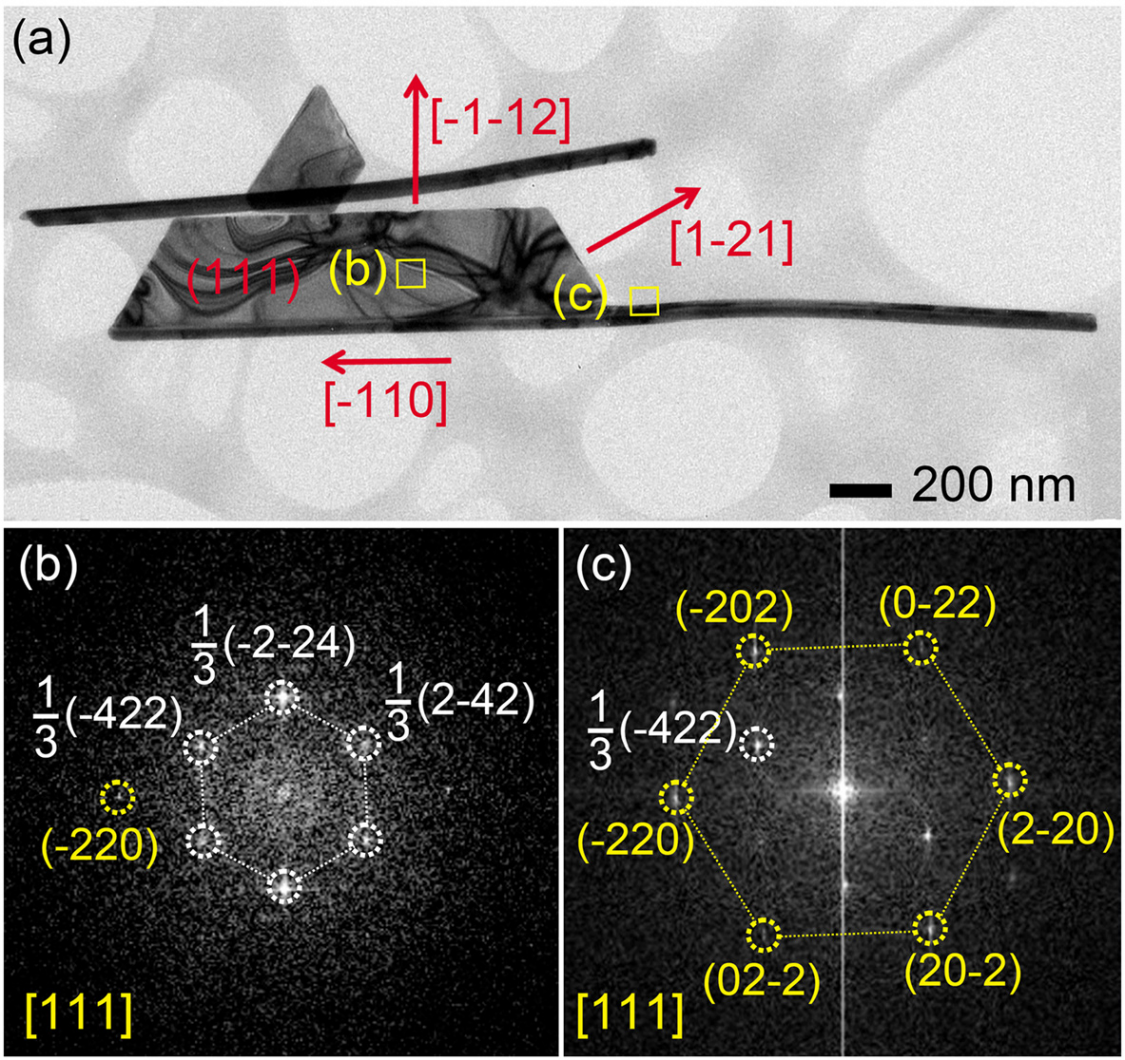
**Crystal structure of Ag nanosheets.** (**a**) BF TEM image of a Ag nanosheet, (**b** and **c**) FFT images of the marked square areas in (**a**), respectively.

Figure 6a shows a bright field (BF) TEM image of Ag nanosheet that was selected from the sample shown in Figure 1a. Ag nanosheet grew along the facetted nanowire, which agreed with the SEM observation. Figure 6b,c shows the fast Fourier transform (FFT) images acquired for the marked areas in Figure 6a. The facetted Ag nanowire had a [−110]-longitudinal direction according to the FFT image of Figure 6c. In the FFT images shown in Figure 6b,c, the inner set of spots might originate from the 1/3{422} planes normally forbidden by an fcc crystal structure. The forbidden 1/3{422} reflections were observed in the nanoplate morphology of Ag or Au due to the stacking faults extending parallel to the {111} planes through the entire nanoplates [9,21,22]. The outer spots were partially indexed to {220} Bragg reflections. The planar surfaces of Ag nanosheet were bounded by {111} planes and the edges were bounded by {112} planes. TEM analyses indicated that the Ag nanosheet was single crystal with {111} planar surfaces bounded by {112} edge planes. The FFT images of the facetted nanowire and the nanosheet showed the same crystallographic direction. This indicated that the nanosheet grew coherently along the facet plane of the nanowire. The present results are similar to the previous results in that gold nanobelts and nanocombs, synthesized in the presence of various organic molecules or surfactants, had grown along the <110> and <211> directions because the mixed surfactants induced anisotropic growth by being adsorbed on specific crystal planes [23,24]. In this study, the filamentary effect in the ultra-dilute concentration, as discussed in the previous work [20], might have induced the strong interface anisotropy needed for the anisotropic planar growth. As the ultra-dilute concentration of electrolyte could bring about a thick double layer between the deposit and the electrolyte [25], the slow transportation of Ag ions to the deposit was being controlled by the reduction potential to enable the facet growth to occur. In addition, different surface energies of each lattice plane might contribute to the anisotropic growth. As the surface energies of {111}, {112}, and {110} planes are known to be 1.6055, 1.8642, and 1.9342 J/m^2^[24,26], it appears that the {111}-planar surface is more favorable thermodynamically.

## Conclusions

We developed a facile, one-step, low-cost, and large-scale method of fabricating single-crystalline Ag nanosheets with controllable thickness without any templates, capping agents, or sacrificial seed materials. The growth of nanosheets occurred in three stages: polygonal island formation, facetted nanowire growth, and planar growth of nanosheet coherent with the facetted nanowire. The nanosheets with {111}-planar surfaces and {112}-edge planes had a controllable thickness depending upon the deposition frequency and reduction/oxidation potentials. The present method is expected to contribute to the development of environment-friendly and low-cost electrochemical synthesis of nanomaterials.

## Competing interests

The authors declare that they have no competing interests.

## Authors' contributions

SHP performed the synthesis of silver nanosheets and drafted the manuscript. JGS and TGL carried out the measurement and analysis of SERS property. HMP contributed to the analysis of the crystal structure of silver nanosheets. JYS initiated and organized the work having the idea of filamentary growth and finalized the manuscript. All authors read and approved the final manuscript.

## References

[B1] Banholzer MJ Millstone JE Qin L Mirkin CA Rationally designed nanostructures for surface-enhanced Raman spectroscopy Chem Soc Rev 2008 8 885 897 10.1039/b710915f 18443674 PMC8207723

[B2] Holt RE Cotton TM Surface-enhanced resonance Raman and electrochemical investigation of glucose oxidase catalysis at a silver electrode J Am Chem Soc 1989 8 2815 2821 10.1021/ja00190a012

[B3] Du J Han B Liu Z Liu Y Control synthesis of silver nanosheets, chainlike sheets, and microwires via a simple solvent-thermal method Cryst Growth Des 2007 8 900 904 10.1021/cg060661t

[B4] Mock JJ Barbic M Smith DR Schultz DA Schultz S Shape effects in plasmon resonance of individual colloidal silver nanoparticles J Chem Phys 2002 8 6755 6759 10.1063/1.1462610

[B5] Maillard M Giorgio S Pileni M-P Tuning the size of silver nanodisks with similar aspect ratios: synthesis and optical properties J Phys Chem B 2003 8 2466 2470 10.1021/jp022357q

[B6] Yang J Qi L Zhang D Ma J Cheng H Dextran-controlled crystallization of silver microcrystals with novel morphologies Cryst Growth Des 2004 8 1371 1375 10.1021/cg049823g

[B7] Feldheim DL Foss CA Jr Metal nanoparticles: synthesis, characterization, and applications 2002 New York: Dekker 150 153

[B8] Liu G Cai W Liang C Trapeziform Ag nanosheet arrays induced by electrochemical deposition on Au-coated substrate Cryst Growth Des 2008 8 2748 2752 10.1021/cg700933p

[B9] Sun Y Metal nanoplates on semiconductor substrates Adv Funct Mater 2010 8 3646 3657 10.1002/adfm.201001336

[B10] Yang S Cai W Kong L Lei Y Surface nanometer-scale patterning in realizing large-scale ordered arrays of metallic nanoshells with well-defined structures and controllable properties Adv Funct Mater 2010 8 2527 2533 10.1002/adfm.201000467

[B11] Zhang W Fischer H Schmid T Zenobi R Martin OJF Mode-selective surface-enhanced Raman spectroscopy using nanofabricated plasmonic dipole antennas J Phys Chem C 2009 8 14672 14675 10.1021/jp9042304

[B12] Dhawan A Zhang Y Yan F Gerhold M Vo-Dinh T Nano-engineered surface-enhanced Raman scattering (SERS) substrates with patterned structures on the distal end of optical fibers Proc SPIE 2008 8 68690G

[B13] Bai J Qin Y Jiang C Qi L Polymer-controlled synthesis of silver nanobelts and hierarchical nanocolumns Chem Mater 2007 8 3367 3369 10.1021/cm0707861

[B14] Liu R Sen A Unified synthetic approach to silver nanostructures by galvanic displacement reaction on copper: from nanobelts to nanoshells Chem Mater 2012 8 48 54 10.1021/cm2017714

[B15] Liu L Yoo S-H Lee SA Park S Electrochemical growth of silver nanobelts in cylindrical alumina nanochannels Cryst Growth Des 2011 8 3731 3734 10.1021/cg2007809

[B16] Chen H Simon F Eychmüller A Large-scale synthesis of micrometer-sized silver nanosheets J Phys Chem C 2010 8 4495 4501 10.1021/jp909206x

[B17] Sun Y Wiederrecht GP Surfactantless synthesis of silver nanoplates and their application in SERS Small 2007 8 1964 1975 10.1002/smll.200700484 17935082

[B18] Liu G Cai W Kong L Duan G Lü F Vertically cross-linking silver nanoplate arrays with controllable density based on seed-assisted electrochemical growth and their structurally enhanced SERS activity J Mater Chem 2010 8 767 772 10.1039/b917167c

[B19] Shin HS Yu J Park HM Song JY Size-dependent lattice parameters of microstructure-controlled Sn nanowires J Mater Res 2011 8 2033 2039 10.1557/jmr.2011.218

[B20] Park SH Shin HS Kim YH Park HM Song JY Template-free and filamentary growth of silver nanowires: application to anisotropic conductive transparent flexible electrodes Nanoscale 2013 8 1864 1869 10.1039/c2nr33056c 23348502

[B21] Germain V Li J Ingert D Wang ZL Pileni MP Stacking faults in formation of silver nanodisks J Phys Chem B 2003 8 8717 8720 10.1021/jp0303826

[B22] Kirkland AI Jefferson DA Duff DG Edwards PP Gameson I Johnson BFG Smith DJ Structural studies of trigonal lamellar particles of gold and silver Proc R Soc Lond A 1993 8 589 609 10.1098/rspa.1993.0035

[B23] Imai H Nakamura H Fukuyo T Anisotropic growth of silver crystals with ethylenediamine tetraacetate and formation of planar and stacked wires Cryst Growth Des 2005 8 1073 1077 10.1021/cg0496585

[B24] Zhao N Wei Y Sun N Chen Q Bai J Zhou L Qin Y Li M Qi L Controlled synthesis of gold nanobelts and nanocombs in aqueous mixed surfactant solutions Langmuir 2008 8 991 998 10.1021/la702848x 18173292

[B25] Zheng X-J Jiang Z-Y Xie Z-X Zhang S-H Mao B-W Zheng L-S Growth of silver nanowires by an unconventional electrodeposition without template Electrochem Comm 2007 8 629 632 10.1016/j.elecom.2006.10.039

[B26] Monk J Hoyt JJ Farkas D Metastability of multitwinned Ag nanorods: molecular dynamics study Phys Rev B 2008 8 024112

